# Reconstructed dose and geometric coverage for tight margins using intrafraction re-planning on an integrated magnetic resonance imaging and linear accelerator system for prostate cancer patients

**DOI:** 10.1016/j.phro.2025.100776

**Published:** 2025-05-10

**Authors:** Ingeborg van den Berg, Cornel Zachiu, Eline N. de Groot-van Breugel, Thomas Willigenburg, Gijsbert H. Bol, Jan J.W. Lagendijk, Bas W. Raaymakers, Harm H.E. van Melick, Cornelis A.T. van den Berg, Jochem R.N. van der Voort van Zyp, Johannes C.J. de Boer

**Affiliations:** a Department of Radiation Oncology, Division of Imaging & Oncology, University Medical Center Utrecht, Utrecht, The Netherlands; b Department of Urology, St. Antonius Hospital, Utrecht/Nieuwegein, The Netherlands

**Keywords:** Intrafraction adaptation, MR-Linac, Magnetic resonance-guided radiotherapy (MRgRT), Prostate cancer (PCa)

## Abstract

**Background and purpose:**

A sub-fractionation workflow enables a substantial reduction in planning target volume (PTV) margin in prostate cancer (PCa) patients by reducing systematic motion during magnetic resonance (MR)-guided radiotherapy. This study assessed geometric and reconstructed dose outcomes in patients treated with a tight-margin sub-fractionation workflow on a combined linear accelerator with a 1.5 T MRI scanner (MR-Linac).

**Materials and methods:**

We evaluated the sub-fractionation workflow with tight margins (2–3 mm) on 128 PCa patients who completed treatment with 5 × 7.25 Gy (36.25 Gy total dose). A traffic light protocol was applied based on residual motions to detect patients with unexpectedly large motions. When ’red’ traffic light criteria were met, plans with larger margins (5 mm isotropic) were adopted for subsequent fractions. Intra- and inter-fraction dose accumulation was performed via an in-house developed deformable image registration algorithm.

**Results:**

A total of 89 % (114/128) of patients completed treatment with the initial tight margins. The mean 3D intrafraction shifts were 1.0 mm (SD: 0.6 mm) in the group with the tight margins and 1.9 mm (SD: 1.5 mm) in the patient group who switched to large margins. The median accumulated D99% was 34.9 Gy (interquartile range: 34.0–35.3 Gy) for patients with prostate shifts who switched to larger margins. In 57 % (8/14) of these patients, the accumulated D99% was above the threshold of 34.4 Gy.

**Conclusions:**

Tight margins of 2–3 mm can be safely applied for at least 95 % (122/128) of the PCa patients undergoing a sub-fractionation workflow on a 1.5 T MR-linac.

## 1 Introduction

Magnetic resonance (MR)-guided radiotherapy in prostate cancer (PCa) patients allows MR imaging before and during treatment delivery [1,2]. The advantage of MR-guided radiotherapy is that it enables adaptation to anatomical changes of the prostate and organs at risk (OARs) through real-time adaptation [3,4]. The clinical outcomes of adaptive radiotherapy for PCa patients show effective and safe delivery and low rates of acute side effects [5]. In recent years, the fractionation schemes for MR-guided radiotherapy have evolved rapidly, embracing hypofractionation with five fractions and even ultra-hypofractionation with two fractions [6,7].

In hypofractionated radiotherapy, longer treatment times and reduced statistical averaging make intrafraction adaptation methods particularly clinically relevant due to the increased risk of higher doses of OARs in case of prostate shifts. Through online adaptation in hypofractionated MRgRT, the coverage of the prostate and seminal vesicles can be improved [8], and the accumulated dose to the OARs can be reduced [9]. Our earlier findings demonstrated that using online plan adaptation without gating, prostate intrafraction motion in MR-guided radiotherapy hinders the feasibility of reducing planning margins below 4 mm [10]. With hypofractionated prostate MRgRT and gating with baseline shift correction, the margins between clinical target volume (CTV) to planning target volume (PTV) can be reduced to 3 mm on a 0.35 T MR-Linac [11,12]. However, despite daily online contour adaptation and re-planning, relevant intrafraction motion of the prostate may still occur due to bladder filling and/or the presence of gas in the rectum during treatment. This primarily leads to intrafraction prostate motion along the superior-interior (SI) and anterior-posterior (AP) direction [10], and potential rotations of the seminal vesicle [13].

Consequently, a new workflow was established that facilitates adaptation to this intrafraction motion by splitting a single fraction dose delivery into two subfractions [14]. This sub-fractionation workflow enables substantial planning target volume (PTV) margin reduction from 5 mm to 2–3 mm in PCa patients by reducing intrafraction motion during MR-guided radiotherapy. In this study, we evaluate the geometric and reconstructed dose outcomes for patients treated using a sub-fractionation workflow with adaptive margins of a traffic protocol on a combined linear accelerator with a 1.5 T MRI scanner (MR-Linac).

## 2 Materials and methods

### 2.1 Sub-fractionation workflow

**Fig. 1:**
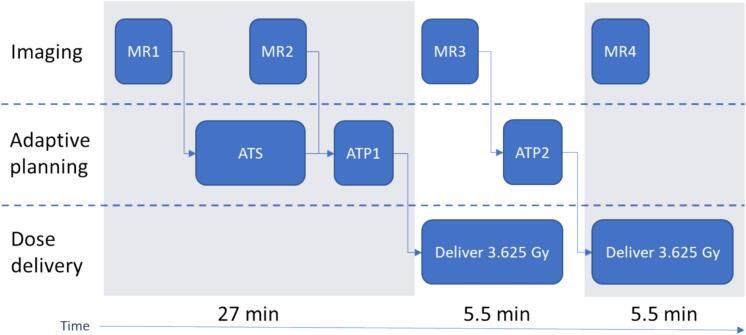
Schematic overview of the sub-fractionation workflow. MR = magnetic resonance. ATS = Adapt-to-Shape. ATP = Adapt-to-Position. The average time from MR1 to ATP1 before the first delivery is 27 min, with the first and second treatment plans of 3.625 Gy each requiring 5.5 min.

We included 128 patients with low-risk or intermediate-risk PCa who were treated on a 1.5 T MR-linac (Unity, Elekta AB, Sweden) between June 2022 and January 2023 with 5 × 7.25 Gy using a sub-fractionation workflow [14]. The clinical target volume (CTV) was defined as the prostate and gross tumor volume (GTV), with a 4 mm isotropic expansion of the GTV (excluding bladder and rectum) to account for microscopic spread. The GTV was determined as the visible tumor observed on multiparametric MRI. The PTV was defined by expanding the CTV by 2 mm in the SI and left–right (LR) directions, and by 3 mm in the AP direction. Each treatment session was split into two subsequent deliveries of 3.625 Gy plans (10 × 3.625 Gy in total). The sub-fractionation workflow detailed in Fig. 1 involves several steps and has a total on-table time of approximately 42.7 min as reported in Willigenburg et al. [14].

First, a daily MRI scan (MR1) was acquired and the Adapt-to-Shape (ATS) procedure was initiated for online contour adaptation and re-planning based on MR1, allowing changes in position, shape, and volume of the target structures. During the ATS procedure, a second MRI scan (MR2) was acquired. The treatment plan of MR1 was adapted to MR2 through the Adapt-to-Position (ATP) procedure (<1 min calculation time) and delivered, allowing a shift in the planned dose distribution. During the delivery of the first plan of 3.625 Gy, a new MRI scan (MR3) was acquired and a second plan with ATP was generated to correct for prostate shifts between MR2 and MR3. The timing of this process allowed the second plan to be prepared for delivery once the first plan had been delivered. Finally, during the delivery of the second plan, a final MRI scan (MR4) was acquired. MR4 was only acquired to analyze the geometric shift and dose delivery in the second plan. The T2-weighted 3D MRI sequence parameters of MR1-MR4 are reported in Supplementary Table S1.

### 2.2 Geometric shift calculations

**Fig. 2:**
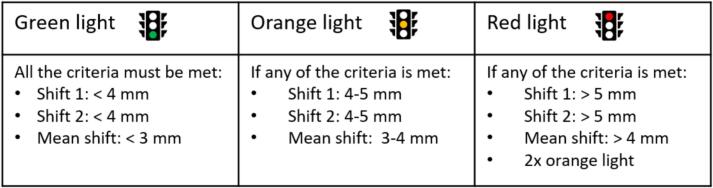
Geometric traffic light protocol in the sub-fractionation workflow.

The residual intrafraction motion was calculated for the sub-fractionation workflow, i.e., the prostate shift between MR3 and MR2 (shift 1), as well as between MR4 and MR3 (shift 2). The residual intrafraction motion was based on the CTV displacement in three translation directions. A traffic light protocol was applied based on these residual intrafraction motions in fractions 1 – 3 to detect patients/fractions with unexpectedly large displacement (Fig. 2). If an ‘orange’ traffic light was encountered in fraction 3, fraction 4 was also monitored. If necessary, a switch to plans with larger margins (typically 5 mm isotropic) for subsequent fractions was made when any of the criteria of the ‘red’ traffic light was met. The tight margins of 2–3 mm were selected because the calculated PTV margins were 1.0 mm in the LR direction, 2.4 mm in the CC direction, and 2.6 mm in the AP direction [14]. The margins criteria of the traffic light protocol were based on systematic errors over the entire treatment with ten subfractions. The tight 2–3 mm margins are not selected as thresholds within the traffic light protocol due to the potential for numerous unwarranted intervention triggers, given the smaller average deviation over 10 shifts compared to within a subfraction or a fraction.

### 2.3 Intrafraction dose calculation

To investigate the delivered dose to the target for those patients who had to switch to larger margins, we investigated the delivered dose to the target, based on all fractions, while also taking into account the intrafraction residue motion. Intra- and inter-fraction dose accumulation was performed via the “Evolution” deformable image registration (DIR) algorithm [15]. Evolution is a variational DIR method that matches similar contrast patterns between two images to estimate any underlying deformations between them. Further details on the cost function and regularization are available in the original study [15]. The algorithm was chosen due to its previously demonstrated capabilities for accurate intra-fraction motion estimation in the scope of image-guided radiotherapy of the prostate [16], as well as for other anatomical locations [17].

In the scope of this work, Evolution was used to register the MR3 and the MR4 scans to the MR1 scan (Fig. 1), with the resulting deformations being used to warp their corresponding ATP1 and ATP2 dose maps. MR3 and MR4 were selected for registration as they were acquired during treatment delivery, which we hypothesized would more accurately reflect the anatomical situation during the delivery of the ATP1 and ATP2 doses, respectively. While delivery of plan ATP1 does not perfectly align with the anatomy in either MR2 or MR3, MR3 was chosen to minimize the risk of under-estimating residual shifts, given its acquisition during ATP1 dose delivery. The resulting warped doses were then added to one another to generate the intra-fraction accumulated dose. The registration and dose accumulation calculations were repeated 30 times, with the median value over the 30 measurements being used for analysis. This ensured representative results by addressing possible minor variations due to Evolution converging towards slightly different local optima. Since our primary focus is on the target dose and the OARs, the relevant dose volume-histogram (DVH) parameters were calculated for the CTV, rectum, and bladder. The target coverage constraint of the CTV for the D99% was 34.4 Gy. The clinical dose constraints for the rectum were set at D1cm^3^ <38.0 Gy and for the bladder at D5cm^3^ <37.0 Gy. The median and the interquartile range (IQR) were calculated for data that was not normally distributed.

### 2.4 Interfraction dose calculation

For the inter-fraction dose accumulation, we employed a specific variation of the Evolution algorithm. Due to the large day-to-day volumetric changes typically undergone by the bladder and rectum, an accurate estimation of their deformations by the original implementation of Evolution is particularly challenging [16]. Therefore, we opted to employ the contour-guided version of the algorithm as described in Bosma et al. [18] and Willigenburg et al. [19]. In our study, the OAR contours were adapted only within a ring structure that extended 2 cm beyond the PTV in the AP and LR directions, and 1 cm in the CC direction. These daily contours of the bladder, prostate, and rectum were used as an additional input to the Evolution algorithm to guide it towards a solution that maximizes its post-registration alignment. This approach was used to register the MR1 scans of fractions 2 – 5 to the MR1 scan of the first fraction. The resulting deformations were used to warp the intra-fraction accumulated dose maps, which were subsequently added to the accumulated dose of the first fraction to determine the overall accumulated dose for the entire treatment.

### 2.5 New dose plans

**Fig. 3:**
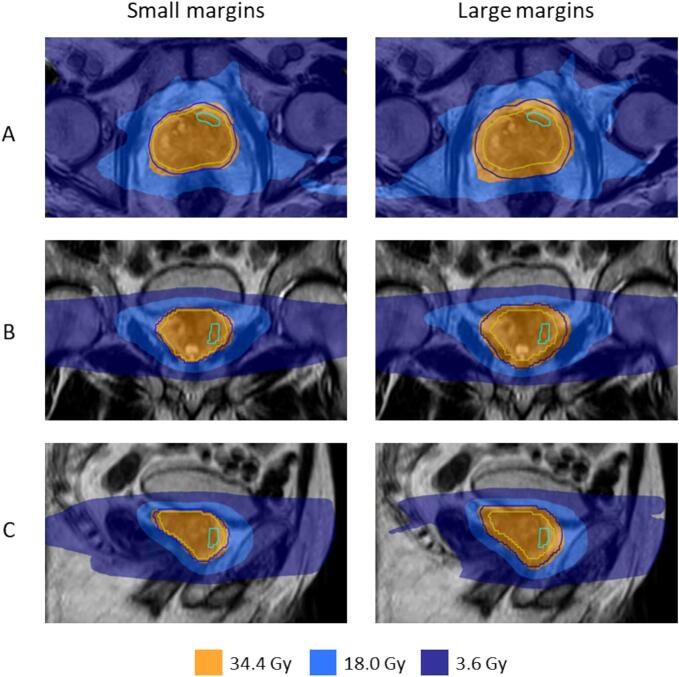
An example of dose plans with small and large PTV margins is shown in three planes: transversal (A), coronal (B), and sagittal (C). The contour of the GTV is in cyan, the CTV is in yellow, and the PTV is in dark blue. (For interpretation of the references to colour in this figure legend, the reader is referred to the web version of this article.)

In a subgroup of patients where a red traffic light occurred, a switch to larger margins was made for subsequent fractions. In this study, these fractions were re-planned with the original tight margins to analyze what the dose of the CTV would have been without applying the traffic light safety measurements. One Radiation Therapy Technologist (RTT) with 16 years of clinical experience generated new dose plans using the online planning software Monaco® (Elekta Inc., Sunnyvale, California, USA). An example of two dose plans with tight and larger margins is shown in Fig. 3.

## 3 Results

### 3.1 Geometric evaluation

**Fig. 4:**
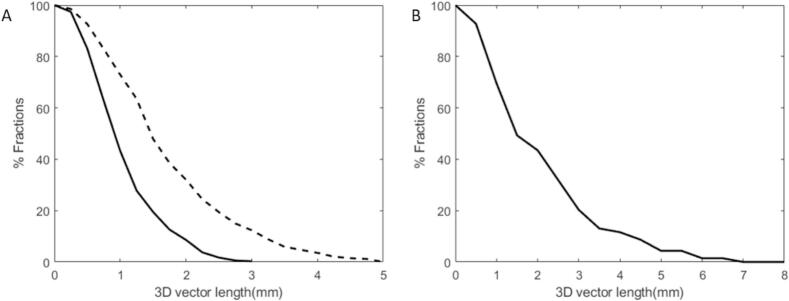
Cumulative distribution of residual 3D intrafraction shifts per fraction. A: Patient group (n = 114) who completed treatment with tight margins. The dashed line shows the 3D vector length when one ATP was given instead of two ATP steps during the sub-fractionation workflow in the patient group who completed treatment with tight margins. B: Patient group (n = 14) with prostate shifts who switched to larger margins.

Treatment was completed by 89 % (114/128) of the patients using the small (2–3 mm) margins. In 11 % (14/128) of the patients, a red traffic light occurred. The cumulative distribution of the residual 3D intrafraction shift per fraction is shown in Fig. 4. The average value of this remaining intrafraction shift over all fractions was 1.0 mm (SD: 0.6 mm) in the patient group with the tight margins and 1.9 mm (SD: 1.5 mm) in the patient group that switched to the larger margins. The intrafraction shift for the patient group who completed treatment with tight margins would have been 1.6 mm (SD: 1.0 mm) if they had been treated with a single ATP using 5 mm margins. The residual systematic errors in the LR, SI, and AP direction were respectively 0.0 ± 0.3 mm, 0.3 ± 0.4 mm, and 0.1 ± 0.4 mm.

### 3.2 Reconstructed dose evaluation

**Fig. 5:**
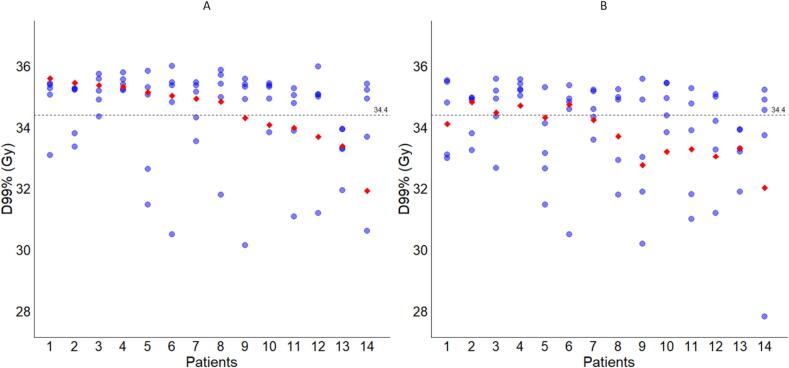
Interfraction D99% doses (blue dots) and fully accumulated D99% doses (red diamonds) for patients with red traffic lights. A: Fraction doses with a shift to larger margins (as clinically applied). B: Re-planned fractions with the original tight margins. The dashed horizontal line corresponds to the prescription dose of 34.4 Gy. (For interpretation of the references to colour in this figure legend, the reader is referred to the web version of this article.)

The intrafraction and accumulated D99% doses of the CTV for the patient group with prostate shifts that triggered a red traffic light are shown in Fig. 5. The intrafraction and accumulated D95% doses of the CTV are shown in Supplementary Fig. 1. The median accumulated CTV D95% and D99% were 35.8 Gy (IQR: 35.5– 35.9 Gy) and 34.9 Gy (IQR: 34.0 – 35.3 Gy), respectively for patients with prostate shifts who switched to larger margins. In 57 % (8/14) of these patients, the accumulated D99% was above the threshold of 34.4 Gy. Patient 14 (Fig. 5) experienced a decrease in the D99% dose in one fraction due to substantial movement caused by urinary urgency. The 3D shift between MR3 and MR4 was 11.7 mm leading to an underdosage at the prostate. A large part of the ATP2 dose was thus delivered to the bladder instead of the prostate. Most patients switched to larger margins in the fourth fraction (6/14), followed by the third fraction (5/14), the fifth fraction (2/14), and the second fraction (1/14). Conversely, when in these patients the original tight margins would have been maintained in subsequent fractions, the median accumulated D99% would be 33.9 Gy (IQR: 33.2 – 34.4 Gy). In this scenario, in 29 % (4/14) of the patients, the target coverage aim of D99% above 34.4 Gy would be met.

**Fig. 6:**
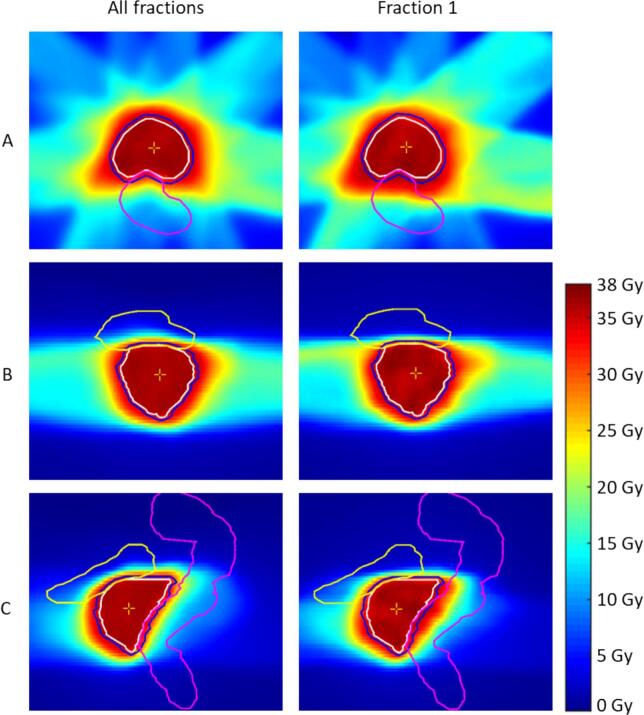
Accumulated dose distributions in axial (A), coronal (B), and sagittal (C) directions for a patient with large geometric shifts who switched to larger margins. Left: Accumulated dose distribution of all fractions. Right: Accumulated dose of fraction 1 with a geometric shift in the anterior-posterior direction. The bladder is visualized in yellow, the rectum in pink, the CTV in white, and the PTV in blue. (For interpretation of the references to colour in this figure legend, the reader is referred to the web version of this article.)

All patients adhered to the clinical dose constraints for the rectum (i.e., D1cm^3^ < 38.0 Gy) and for the bladder (i.e., D5cm^3^ < 37.0 Gy). The accumulated doses in the rectum and bladder are represented in Supplementary Table S2. In the patient group with large geometric shifts, the median accumulated D1cm^3^ for the rectum was 35.0 Gy (IQR: 34.1 – 35.7 Gy) and the median accumulated D5cm^3^ for the bladder was 32.5 Gy (IQR: 30.1 – 34.2 Gy). If the tight margins had been maintained across all fractions, the accumulated D1cm^3^ for the rectum would be 34.2 Gy (IQR: 33.0 – 34.7 Gy) and the median accumulated D5cm^3^ for the bladder would be 31.1 Gy (IQR: 28.8 – 33.7 Gy). An example of the dose distribution for one patient with large geometric shifts in the AP direction during the first fraction (Patient 5 in Fig. 5) is shown in Fig. 6.

## 4 Discussion

This study presents the geometric and reconstructed dose outcomes for patients treated using a sub-fractionation workflow with 2–3 mm tight margins on a 1.5 T MR-Linac. The geometric results confirm that tight margins can be safely applied with the sub-fractionation workflow in at least 95 % (122/128) of the patients. We expect this percentage to grow further with more loose traffic light criteria based on increasing experience. Fourteen patients with a red traffic light switched to larger margins in the subsequent fractions. Retrospective analysis using a dose accumulation over all five fractions showed that without margin enlargement, the target CTV coverage (D99%) would not have been met in 71 % (10/14) of these patients. Increasing the margins improved target coverage in nine out of ten patients but restored the target coverage in just four out of ten patients. In 21 % (3/14) of the patients, the accumulated dose was slightly below the 34.4 Gy threshold, and larger margins would likely not have been necessary. The OAR constraints for the rectum and bladder were met in the complete patient group, even in patients with large geometric shifts.

A margin calculation that included other remaining uncertainties (e.g., intrafraction rotation) confirmed the validity of the applied tight margins. The mean 3D intrafraction shifts were 1.0 mm (SD: 0.6 mm) in the patient group with tight margins. Our results are comparable to those found in the study by Willigenburg et al. [14], which reported a mean 3D intrafraction shift of 1.1 mm (SD: 0.7 mm) in other patients treated using the same sub-fractionation workflow on a 1.5 T MR-Linac. The mean 3D intrafraction shifts for a standard 7.25 Gy plan delivery, which uses one ATP step rather than two ATP steps in the sub-fractionation workflow, would be 1.6 mm (SD: 1.1 mm) for the lower limit and 2.3 mm (SD: 1.4 mm) for the upper limit [14]. The residual systematic errors in this study were highest in the SI direction with 0.3 mm due to differences in bladder filling, followed by 0.1 mm in the AP direction, and 0.0 mm in the LR direction. One patient experienced urinary urgency, leading to a significant geometric shift, and subsequently resulting in an underdosage to the prostate. Intrafraction motion is difficult to predict [20], making it valuable to explore whether planning MRI could aid in its prediction. For instance, Ballhausen et al. [21] found that a larger inner diameter of the lesser pelvis (i.e., the lateral distance between the two pubic bones) on a planning CT scan can predict higher prostate intrafraction motion. Identifying anatomical predictors could support the adoption of looser traffic light criteria by helping to identify patients for whom tight margins remain appropriate.

In patients with accumulated CTV doses below the 34.4 Gy threshold, a sub-fractionation workflow does not address significant geometric shifts during the second dose delivery. While delivering the first plan of 3.625 Gy, an MRI scan (MR3) was acquired, and a second plan was generated to correct prostate shifts. However, if the final MRI scan (MR4) acquired during the second plan delivery of 3.625 Gy reveals larger prostate shifts, these are not corrected. Retrospective analysis showed that eight patients experienced shifts of more than 5 mm during the second delivery, with even two patients having more than 10 mm shifts. We chose to implement the traffic light protocol with larger margins in all subsequent fractions to maximize the probability of adequate dosimetric coverage of the CTV.

In the patient group with large geometric deviations, gating and intrafraction drift correction can hold great potential. The Comprehensive Motion Management (CMM) on a 1.5 T MR-Linac (Unity, Elekta AB, Sweden) has been introduced and recently evaluated for patients with abdominal lesions [22]. This active motion management enables automatic gating of the radiation beam and drift correction with baseline shifting based on real-time cine MRI target tracking [23,24]. An in silico evaluation of intrafraction drift correction demonstrated DVH metrics comparable to those of the sub-fractionation workflow for mid-treatment adaptation [25]. Moreover, for patients with minor prostate shifts who do not require any drift correction, active motion management is beneficial. This enables faster treatment delivery without the need for sub-fractionation. There are some limitations associated with this study. First, we included the DVH parameters of the bladder and rectum but the clinical ATS and ATP contours were only corrected within a 1–2 cm ring around the target structure. We consider the high dose-volume values (e.g., D1cc) in the accumulated doses for OAR more reliable than low values because both the bladder and rectum show more intrafraction deformation than the prostate. However, bladder and rectum volumes close to the prostate are still reliable. Implementing deep learning-based auto-contouring for segmentation and registration of OARs [26,27] might improve the accuracy of OARs delineation and reduce the need for manual adjustments both inside and outside the ring structure. Second, we evaluated the geometric shift over the complete patient group but we did not perform the reconstructed dose evaluation for the PCa patients with small geometric shifts (green traffic light). We demonstrated that the target coverage was clinically reached in this group in a separate study [28]. Third, the evaluation of our study is only applicable to the sub-fractionation workflow as a motion management technique for PCa patients.

In conclusion, tight margins of 2–3 mm can be safely applied when using a sub-fractionation workflow in at least 95 % of the PCa patients treated on an MR-Linac. The sub-fractionation workflow demonstrated low mean 3D intrafraction shifts of 1.0 mm in the patient group with tight margins and 1.9 mm in the patient group that switched to larger margins. Future research should enhance geometric and reconstructed dose evaluation of gating and intrafraction drift correction for PCa patients, enabling potentially better corrections for patients with large geometric shifts.

## CRediT authorship contribution statement

**Ingeborg van den Berg:** Conceptualization, Formal analysis, Investigation, Methodology, Visualization, Writing – original draft, Writing – review & editing. **Cornel Zachiu:** Conceptualization, Methodology, Software, Writing – review & editing. **Eline N. de Groot-van Breugel:** Formal analysis, Writing – review & editing. **Thomas Willigenburg:** Writing – review & editing. **Gijsbert H. Bol:** Writing – review & editing. **Jan J.W. Lagendijk:** Writing – review & editing. **Bas W. Raaymakers:** Writing – review & editing. **Harm H.E. van Melick:** Writing – review & editing. **Cornelis A.T. van den Berg:** Writing – review & editing. **Jochem R.N. van der Voort van Zyp:** Writing – review & editing. **Johannes C.J. de Boer:** Conceptualization, Methodology, Visualization, Supervision, Writing – review & editing.

## Declaration of competing interest

The authors declare that they have no known competing financial interests or personal relationships that could have appeared to influence the work reported in this paper.
