# Supplementary material for: Reconstructed dose and geometric coverage for tight margins using intrafraction re-planning on an integrated magnetic resonance imaging and linear accelerator system for prostate cancer patients
Source: Phys Imaging Radiat Oncol. 2025 May 10;34:100776. doi: 10.1016/j.phro.2025.100776 (PMC12145722; doi:10.1016/j.phro.2025.100776)
Supplement: Supplementary Data 1 [file mmc1.docx]

**Supplementary Materials**

**
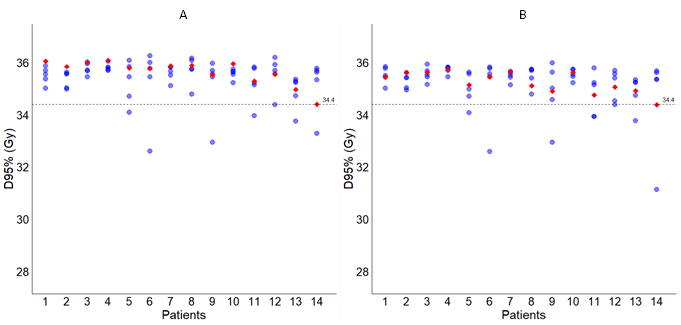
**

**Supplementary Figure S1.** Interfraction and fully accumulated D95% doses for patients with red traffic lights. A: Fraction doses with a shift to larger margins. B: Re-planned fractions with the original tight margins.

**Supplementary Table S1: MRI parameters.**

| **Parameter** |  |
| --- | --- |
| Field of View | 400x400x180 mm or 400x447x180 mm, depending on patient size |
| Acquisition voxel size | 1.5x1.5x2.0 mm |
| Reconstructed voxel size | 0.78x0.78x2.0 mm |
| Repetition time | 1635 ms |
| Echo time | 120 ms |
| Flip angle | 90 degrees with constant refocusing control of 100 degrees |
| Acquisition time | 3 min 05 sec or 3 min 24 sec, depending on patient size |

**Supplementary Table S2: Accumulated doses in rectum and bladder in the patient group with a ‘red’ traffic light.**

|  | Rectum | Bladder |
| --- | --- | --- |
| D0.5cc | 35.6 Gy (34.9 – 36.1) | 35.9 Gy (35.5 – 36.6) |
| D1cc | 35.0 Gy (34.1 – 35.7) | 35.6 Gy (34.5 – 36.3) |
| D2cc | 34.1 Gy (32.7 – 34.8) | 35.0 Gy (33.1 – 35.8) |
| D5cc | 30.9 Gy (28.8 – 31.9) | 32.5 (30.1 – 34.2) |

*Data is presented as median (interquartile range). The accumulated doses are presented in the patient group (n = 14) where a ‘red’ traffic light occurred and a switch to larger margins (5 mm isotropic) was made for subsequent fractions. The clinical dose constraints for the rectum were set at D1cc < 38.0 Gy and for the bladder at D5cc < 37.0 Gy.
